# The relationship between cognitive reserve focused on leisure experiences and cognitive functions in bipolar patients

**DOI:** 10.1016/j.heliyon.2023.e21661

**Published:** 2023-11-01

**Authors:** Kuniko Sato, Mie Matsui, Yasuki Ono, Yoshiaki Miyagishi, Makoto Tsubomoto, Nobushige Naito, Mitsuru Kikuchi

**Affiliations:** a Laboratory of Clinical Cognitive Neuroscience, Graduate School of Medical Science, Kanazawa University, Kakuma-machi, Kanazawa, Ishikawa 920-1192, Japan; b Laboratory of Clinical Cognitive Neuroscience, Institute of Liberal Arts and Science, Kanazawa University, Kakuma-machi, Kanazawa, Ishikawa 920-1192, Japan; c Department of Neuropsychiatry, Graduate School of Medicine, Hirosaki University, 1 Bunkyocyo, Hirosaki, Aomori 036-8224, Japan; d Department of Psychiatry and Behavioral Science, Kanazawa University Graduate School of Medicine, 13-1 Takara-machi, Kanazawa 920-8641, Japan

**Keywords:** Cognitive function, Cognitive reserve, Leisure experiences, Bipolar disorder

## Abstract

Bipolar disorder (BP) is characterized by cognitive decline. Individual differences exist in maintaining cognitive function due to daily physical activity and sleep. We examined the relationship between leisure experiences as proxies for cognitive reserve (CR) and cognitive function in patients with bipolar disorder after adjusting for daily physical activity and sleep.

The CR of patients with BP (n = 24) and healthy study controls (HC) (n = 24) was assessed using premorbid IQ, years of education, and leisure activity history. Performance-based neuropsychological tests were performed to evaluate cognitive function. A self-reported scale was used to assess resilience. Physical activity and sleep were measured using an activity meter.

Verbal fluency, story memory, and verbal memory were significantly positively correlated with the kinds of leisure experiences in patients with BP. A hierarchical regression analysis accounting for confounding factors showed that verbal fluency and memory were associated with the kinds of leisure experiences. Neither years of education nor resilience were significantly associated with neuropsychological scores.

Various leisure experiences in patients with BP are associated with higher language-related cognitive functioning. Engaging in various leisure experiences may affect higher cognitive functions related to language.

## Highlights

• Verbal fluency was associated with the kinds of leisure experiences in BP patients.
• Verbal memory was associated with the kinds of leisure experiences in BP patients.
• A variety of leisure experiences may be related to the cognitive function of BP.

## 1 Introduction

Previous reports have shown that 27.0 % of BP patients were found to have severe cognitive impairment, and 21.8 % have good cognitive function [1]. Furthermore, BP patients are impaired across various cognitive domains [2]. Deficits in cognitive measures of verbal learning and memory, visual memory, sustained attention, processing speed, verbal fluency, set-shifting, working memory, response inhibition, visuospatial ability, and general intelligence have been observed in patients with BP [3]. Among patients with BP in remission, the percentage of patients with cognitive impairment ranged from 5.3 to 57.7 % in executive function, 9.6–51.9 % in attention/working memory, 23.3–44.2 % in speed/reaction time, 8.2–42.1 % in verbal memory, and 11.5–32.9 % in visual memory [4]. Additionally, longitudinal assessments of patients with BP have shown no progressive cognitive decline in verbal memory, visual memory, processing speed, sustained attention, executive function, verbal fluency, or working memory [5]. BP has subtypes—type I, type II, and mixed—according to the Diagnostic and Statistical Manual of Mental Disorders-5 (DSM-5), and mood states fluctuate over time [6]; hence, cognitive dysfunction due to BP has been described as complex and heterogeneous. The fact that these impairments persist over many years, affecting learning, work, and family life, emphasizes the need to identify the sociodemographic and clinical factors significantly associated with cognitive impairment in BP patients [4].

CR postulates that individual differences in cognitive processes or neural networks underlying task performance allow some people to cope better than others with brain damage [7]. CR can potentially reduce cognitive decline despite its effects on brain pathology and aging [7]. CR, defined as achievement in years of education, has been reported to protect against cognitive decline, indicating that premorbid intelligence acts on brain pathology as a neural compensation [8,9]. CR is among the most notable predictors of cognitive and functional outcomes in BP patients [10]. CR is also associated with cognitive and psychosocial functioning in patients with bipolar disorder [11]. CR was calculated based on three variables: years of education, estimated IQ, and frequency of reading and writing habits, which have been reported to positively influence executive function in patients with bipolar disorder [12]. On the other hand, from a multidimensional perspective, higher competence in independent living, a skilled profession, and better use of leisure time have been reported to be significantly associated with better performance in planning, set-shifting, and fluency tasks in BP [13].

Another factor that may influence cognitive function in patients with BP is resilience [14]. Resilience, defined as “positive adaptation in the face of significant adversity,” has been associated with social functioning in BP patients [[15], [16], [17]]. Psychological resilience is strongly associated with subjective cognitive functioning [18]. In contrast, no significant relationship exists between resilience and cognitive function in BP [14].

However, psychosocial factors (such as cognitive reserve and resilience) and daily behaviors (such as daily physical activity and sleep) may affect cognitive function in patients with BP. Lifestyle factors such as physical activity and sleep may contribute to CR and attenuate the damaging effects of brain changes on cognition [19]. Indeed, it has been reported that patients were a sedentary diagnostic subgroup and that physical activity and sedentary behavior should be considered [20]. BP patients with mania and depression interspersed with periods of remission have been shown to have longer periods of depression than patients with mania alone [21]. Depression in patients with BP and sedentary behavior is associated with reduced activity [22,23]. It is unclear how different aspects of sleep are associated with cognitive function in studies with large sample sizes [24]. In several studies, physical activity and exercise have been shown to improve cognitive function in older adults [25,26].

Based on these findings, we hypothesized that CR enhances the maintenance of cognitive function as a potential protective factor for patients with BP, even after accounting for daily behaviors such as daily physical activity and sleep conditions. Therefore, this pilot study aimed to identify potential protective factors for cognitive function in patients with BP. This was done by analyzing the associations between cognitive function and CR, focusing on leisure experience while adjusting for daily physical activity and sleep conditions, and using an accelerometer in patients with BP and HC.

## 2 Methods

### 2.1 Participants

The participants were the 24 outpatients or inpatients with BP at the Department of Neuropsychiatry, Kanazawa University Hospital, and the 24 healthy study controls (HC) from July 2019 to July 2021. The patient group (BP) was in remission or had mild depression as diagnosed with BP II by a psychiatrist according to the DSM-5 criteria [27]. The HC group was adjusted to match the BP group in terms of mean age and sex ratio. Written informed consent was obtained from all patients before participation in the study. The present study was conducted according to the Declaration of Helsinki, and the protocol was approved by the Ethics Committee of Kanazawa University (No. 2018–217).

### 2.2 Assessments

#### 2.2.1 Clinical symptoms evaluation

For demographic assessment, in addition to information on age, sex, weight, and height, the Beck Depression Inventory (BDI) (0–63 points) [28] was used to evaluate the degree of depressed mood in HC. For the clinical assessment of BP, the level of depression was assessed using the Hamilton Rating Scale for Depression (HRSD) (0–65 points) [29]. The level of manic state was assessed by structured interviews using the Young Mania Rating Scale (YMRS) (0–60 points) [30]. Daily doses of antipsychotic medication were assessed using chlorpromazine equivalents [31].

#### 2.2.2 Cognitive reserve variables

CR proxies were estimated using premorbid IQ, years of education, and history of leisure experiences. Premorbid IQ was estimated using the Japanese Adult Reading Test [32]. Leisure experiences covered 98 leisure experience categories drawn from official surveys on leisure experiences prepared by the Japanese government and from previous studies in Japan [33,34]. The 98 leisure experiences included physical activities, such as sports; social activities, such as volunteering; and hobbies, such as watching movies and reading. Participants were asked whether they had regularly engaged in each of the 98 leisure experience categories for at least one year, either in the past or present. The total kinds of leisure experience categories in which each individual participated were used as the rating (0–98 points) (Table 1).

**Table 1:** Leisure experiences.

Code	Leisure experiences	Code	Leisure experiences
1	Admiring arts	50	Listening to a radio
2	Going to theaters	51	Hanging out with friends
3	Going to movie theaters	52	Going out, dating
4	Watching movies at home	53	Joining public events
5	Going to concerts	54	Looking after pets
6	Listening to music	55	Relaxing, taking a nap, getting a massage
7	Playing the piano	56	Watching sports
8	Playing string instruments	57	Playing baseball, softball
9	Playing wind instruments	58	Playing volleyball, beach volleyball
10	Playing percussion instruments	59	Playing basketball
11	Playing traditional Japanese instruments	60	Playing football, soccer
12	Playing other imstruments	61	Playing tennis
13	Playing in a band	62	Playing badminton
14	Singing in a chorus group	63	Playing table tennis
15	Going to Karaoke	64	Playing golf
16	Japanese dancing	65	Playing other sports
17	Dancing, bollroom danging, ballet	66	Martial arts
18	Calligraphy	67	Other fighting sports
19	Flower arrangement	68	Playing rugby
20	Tea ceremony	69	Bowling
21	Handcrafting, knitting	70	Fishing
22	Cooking, baking	71	Swimming
23	Gardening	72	Scuba Diving, Snorkelling
24	Growing vegitables	73	Other water sports
25	Home carpentry	74	Skiing, Snowboarding
26	Drawing	75	Mountaineering, Hiking
27	Sclupting	76	Cycling
28	Photography	77	Jogging, Marathon, Running
29	Creative writing	78	Walking
30	Reading books	79	Going for a walk, Picnic
31	Reading comics	80	Exercising
32	Playing traditional Japanese bored games (Shogi, Go)	81	Working out
33	Pachinko	82	Yoga
34	Race betting	83	Domestic travels
35	Lottery	84	International travels
36	Investing	85	Visiting hometowns
37	Playing Mah-jongg	86	Travelling for sightseeing, visiting hot springs
38	Playing video games	87	Going for a drive, motorcycle ride
39	Playing borad games and card games with others	88	Online shopping
40	Playing puzzle or quiz games alone	89	Using electronics devices for entertainmant
41	Visiting touristic facilities (zoos, museums, aquariums, etc.)	90	Using SNS, playing online games
42	Visiting religious buildings	91	Other internet use
43	Camping	92	Studying foreign languages
44	BBQ	93	Using electronics devices for study or programming
45	Evening drink	94	Attending to buisiness lessons
46	Eating out	95	Attending to lessons
47	Shopping	96	Studying for qualifications
48	Information collection and shopping related to beauty and fashion	97	Volunteer works
49	Watching a progam on TV	98	Joining neighbourhood activities

#### 2.2.3 Assessment of resilience

Resilience was assessed using a resilience scale (RS) (25, 175 points) [35,36]. The scale measures an individual's resilience, and each item is scored on a 7-point scale from (1 = completely disagree) to (7 = completely agree). Higher scores indicate higher levels of resilience. An example item is “I can overcome difficulties.” The reliability and validity of the RS have been tested in various populations [35]. This scale has also been used in studies of clinical and biological correlates of resilience in BP patients [37].

#### 2.2.4 Assessment of cognitive functioning

All participants completed performance-based neuropsychological tests to evaluate their cognitive function, including executive function, attention and processing speed, verbal fluency, story memory, and verbal memory.

#### 2.2.5 Executive function

The Wisconsin Card Sorting Test (WCST) [38], assessed executive function. The participants were required to classify the cards given to them by an examiner. Participants classify the cards as “right” or “wrong.” The number of accomplishments completed and errors made when implementing the six categories were scored.

#### 2.2.6 Attention and processing speed assessment

Trail Making Test Part A (TMT - A) assesses processing speed [39]. The TMT-A is a task in which numbers are linked in ascending order with a line drawn from one numbered circle to the next; the numbers in the paper are arranged from 1 to 25. The time taken to link all numbers was recorded. The Symbol Digit Modalities Test (SDMT) assesses processing speed [40]. The SDMT was scored by correctly matching numbers corresponding to the nine symbols according to the key at the top of the page within the allotted time (120 s).

#### 2.2.7 Verbal fluency

The Japanese version of the Verbal Fluency Task (VFT) measures language function [41]. Participants were asked to generate as many words as possible within 60 s. We implemented five categories: words beginning with “ka” and “ta” and categories of animals, fruits, and vegetables. The total number of words generated in each category is used as the score.

#### 2.2.8 Story memory/Verbal memory

The story recall subtest in the Japanese version of the Rivermead Behavioural Memory Test (RBMT) examines episodic memories [42]. The number of sentences recalled immediately was scored (score range: 0–25). The Japanese Verbal Learning Test (JVLT) assesses short-term verbal retention and verbal learning abilities [43]. The JVLT comprises 16-word lists (four categories × four words). The participants were required to recall the word lists after the examiners finished presenting them. The JVLT has three trials; the scores given are the sum of the number of words correctly recalled in each trial (score range: 0–48).

### 2.3 Assessment of daily activity patterns

The BP and HC groups were fitted with activity meters (MTN - 220) (SleepSign Act Ver. 2.) for five days. After setting the age, sex, height, and weight of the participants, the device automatically calculated total sleep time, sleep latency (the time between entering the bed and falling asleep), and sleep efficiency (the ratio of actual sleep time to the time spent lying in bed). Activity meters automatically calculated the amount of physical activity in six directions (FS - 770, MTN - 220) (LifeCorder GS). Total sleep time (minutes/day), sleep latency (minutes/day), sleep efficiency (%), and were calf larger the/day each individual per day based on these calculated values. Patients received CR, resilience assessment, and cognitive function tests during hospitalization immediately before discharge. After discharge, patients with BP were fitted with an activity meter to measure their daily activities.

### 2.4 Statistical analysis

All data were analyzed using IBM SPSS Statistics for Windows version 27 (IBM). Basic descriptive statistics included mean (*M*), standard deviation (*SD*), number of samples (n), and percentage (％) of continuous data. Pearson's correlation coefficient assessed the association between CR, resilience, and cognitive function. Six cognitive functions were examined in both the BP and HC groups. We used the Bonferroni correction for the significance level of variable correlations; *p* < 0.05/24 = 0.002 indicated statistical significance in the analysis. Next, hierarchical regression analysis was conducted to examine the relationship between the variables extracted from the analysis and only those for which significant correlations were found. In the hierarchical regression analysis, the variables extracted in the first step were considered before the dependent variables. The variables from Steps 1 to 3 were entered as independent variables. Age, sex, illness duration, and depression were entered in Step 1. Step 2 was followed by total sleep time and physical activity, obtained from activity meters. In Step 3, resilience, IQ before illness, years of education, and the kinds of leisure experience types —thought to have protective effects on the cognitive function of BP patients—were entered sequentially. Steps 1 and 2 were confounders. Hierarchical regression analysis corrected for Bonferroni correction by examining the three cognitive functions; *p* < 0.05/3 = 0.017 was considered statistically significant.

## 3 Results

### 3.1 Clinical evaluation and cognitive function

The age range of the patients was 18–65 years (mean 43.3 ± 14.3 S.D.). The age range of the 24 healthy study controls with no history of mental illness was 19–65 years (mean 42.8 ± 15.0 S.D.). The medications used in the patient group included lithium (n = 11, 45.8 %), quetiapine (n = 5, 20.8 %), and olanzapine (n = 3, 12.5 %). Table 2 shows no significant differences between the BP and HC groups following the *t*-test for mean values of Body Mass Index (BMI), premorbid IQ, years of education, and history of leisure experiences. The BP group scored significantly lower (*t*_(46)_ = −3.43, *p* = 0.001) than the HC group on mean resilience.

**Table 2:** Demographics of bipolar disorder patients and healthy controls.

	Bipolar disorder patients (*n* = 24)		Healthy controls (*n* = 24)		Student's t-tests	
Male/Female (*n*)	6	/18	6	/18		
Variables	*M*	*SD*	*M*	*SD*	*t-value*	*p*
Age (years)	43.3	14.3	42.8	15.0	0.13	0.899
BMI	24.1	5.4	21.6	2.9	1.96	0.057
Cognitive reserve proxies						
IQ	103.5	8.3	107.2	6.4	−1.75	0.087
Education (years)	14.6	2.7	16.0	12.4	−1.96	0.057
Leisure	15.9	4.3	18.6	6.2	−1.79	0.080
RS	103.0	23.4	123.5	17.8	−3.43	0.001**
Clinical variables						
BDI	–	–	5.0	4.3		
Age at onset (years)	34.1	11.5	–	–		
Duration of illness (years)	8.4	6.2	–	–		
HRSD	9.1	5.5	–	–		
YMRS	2.1	3.6	–	–		
Cognitive function						
WCST (total errors)	28.8	21.4	10.5	5.6	4.03	<0.001**
TMT-A (seconds)	48.6	14.9	32.0	9.8	4.55	<0.001**
SDMT (raw score)	73.9	19.7	96.9	15.4	−4.52	<0.001**
VFT (total number of all scores)	66.4	18.5	76.3	14.0	−2.08	0.043*
RBMT (number of syllables)	11.3	4.6	15.2	3.2	−3.39	0.001**
JVLT (total number of words)	28.4	11.1	36.6	6.6	−3.12	0.003**
Daily activities						
Total sleep time (minutes/day)	389.2	116.4	329.5	68.7	2.17	0.036*
Sleep latency (minutes/day)	19.8	14.1	16.5	8.9	0.99	0.328
Sleep efficiency (％)	78.1	8.8	79.0	9.1	−0.35	0.729
Physical activity (kcal/day)	363.5	241.8	368.7	127.4	−0.09	0.927

Notes: ⁎ *p* < 0.05; ⁎⁎ *p* < 0.01, M = Mean, SD = Standard Deviation, BMI = Body Mass Index, IQ = IQ by Japanese Adult Reading Test, Leisure = Kinds of types of leisure experiences, RS = Resilience Scale, BDI = Beck Depression Inventory, HRSD = Hamilton Rating Scale for Depression, YMRS = Young Mania Rating Scale for Manic Symptoms, WCST = Wisconsin Card Sorting Test, TMT-A = Trail Making Test A, SDMT = Symbol Digit Modalities Test, VFT = Verbal Fluency Test, RBMT = Rivermead Behavioural Memory Test, JVLT = Japanese Verbal Learning Test, Sleep latency = the time required for falling into a deep, Sleep efficiency = the ratio of the total time spent asleep (sleep duration ÷ bed duration Sleep duration).

The results of comparing cognitive function and daily activities between the BP and HC groups are presented in Table 1. For cognitive function, the mean values of executive function, working memory, attention and processing speed, verbal fluency, and story and verbal memory were significantly lower in the BP group than in the HC group (*p* < 0.01) for all tests. Regarding daily activities, the mean total sleep time was significantly higher in the BP group than in the HC group (*t*_(46)_ = 2.17, *p* = 0.036). The mean sleep latency, efficiency, and physical activity were not significantly different between the BP and HC groups. In the *t*-test for two independent groups, effect size, α error probability, and power were set at 0.5, 0.05, and 0.8, respectively. The total sample size and actual power were 26 and 0.806, respectively.

### 3.2 Correlation between cognitive function outcomes, cognitive reserve variables, and RS outcomes

Pearson's test results are presented in Table 3, Table 4. In the BP group, there were significant positive correlations between the kinds of leisure experiences and VFT (*r*_(22)_ = 0.757, *p* < 0.01), RBMT (*r*_(22)_ = 0.605, *p* < 0.01), and JVLT (*r*_(22)_ = 0.583, *p* < 0.01). The BP group had no significant correlations between these cognitive functions and pre-morbid IQ scores, education, or resilience. The HC group had no significant correlations between these cognitive functions and the premorbid IQ, education, leisure, or resilience scores.

**Table 3:** Pearson's correlation coefficient between cognitive function outcomes and variables of Cognitive reserve and RS outcomes in patients with bipolar disorders.

Variables	WCST	TMT-A	SDMT	VFT	RBMT	JVLT
IQ	−0.138	−0.170	0.391	0.195	0.229	0.478
Education	0.013	−0.157	−0.079	0.039	0.143	0.025
Leisure	0.426	−0.545	0.386	0.757*	0.605*	0.583
RS	−0.019	−0.213	0.286	0.187	0.218	0.034

Notes: ⁎ *p* < 0.002, WCST = Wisconsin Card Sorting Test, TMT-A = Trail Making Test A, SDMT = Symbol Digit Modalities Test, VFT = Verbal Fluency Test, RBMT = Rivermead Behavioural Memory Test, JVLT = Japanese Verbal Learning Test, IQ = IQ by Japanese Adult Reading Test, Leisure = Kinds of types of leisure experiences, RS = Resilience Scale.

**Table 4:** Pearson's correlation coefficient between cognitive function outcomes and variables of Cognitive reserve and RS outcomes in healthy controls.

Variables	WCST	TMT-A	SDMT	VFT	RBMT	JVLT
IQ	0.261	−0.290	0.139	0.290	0.189	−0.087
Education	−0.013	0.311	−0.035	0.061	−0.035	−0.120
Leisure	−0.253	−0.310	0.083	0.214	−0.346	−0.017
RS	0.186	0.014	−0.027	0.068	−0.064	−0.131

Notes: ⁎ *p* < 0.002, WCST = Wisconsin Card Sorting Test, TMT-A = Trail Making Test A, SDMT = Symbol Digit Modalities Test, VFT = Verbal Fluency Test, RBMT = Rivermead Behavioural Memory Test, JVLT = Japanese Verbal Learning Test, IQ = IQ by Japanese Adult Reading Test, Leisure = Kinds of types of leisure experiences, RS = Resilience Scale.

### 3.3 Hierarchical regression analysis with bipolar disorder patients

Hierarchical regression analysis was conducted to examine the associations between CR and resilience using the VFT, RBMT, and JVLT in the BP group. As shown in Table 5, the hierarchical regression model revealed that the kinds of leisure experience types in the final step were positively associated with the VFT. For the RBMT, as shown in Table 6, no significant associations were found for any of the steps. As shown in Table 7, in the final step, the kinds of leisure experience types and premorbid IQ scores were positively associated with the JVLT.

**Table 5:** Results of hierarchical regression analysis with bipolar disorder patients in VFT.

Variables	VFT		
	β		
	Step1	Step2	Step3
Age	−0.040	−0.317	0.099
Sex	0.330	0.464	0.246
Duration of illness	−0.221	0.020	−0.039
HRSD	−0.179	0.002	0.048
Total sleep time		−0.364	−0.295
Physical activity		0.370	0.329
RS			−0.140
IQ			0.306
Education			−0.151
Leisure			0.729*
**Goodness of fit of the model**			
*Adjusted R*^*2*^	0.003	0.193	0.686
*df*	19	17	13
*p*	0.442	0.136	0.002

Notes: ⁎ *p* < 0.017. VFT = Verbal Fluency Test, HRSD = Hamilton Rating Scale for Depression, IQ = IQ by Japanese Adult Reading Test, RS = Resilience Scale, Leisure = Kinds of types of leisure experiences.

**Table 6:** Results of hierarchical regression analysis with bipolar disorder patients in RBMT.

Variables	RBMT		
	β		
	Step1	Step2	Step3
Age	−0.185	−0.418	−0.080
Sex	0.278	0.444	0.296
Duration of illness	−0.210	−0.011	−0.154
HRSD	0.120	0.236	0.283
Total sleep time		−0.224	−0.130
Physical activity		0.405	0.241
RS			0.140
IQ			0.254
Education			−0.022
Leisure			0.426
**Goodness of fit of the model**			
*R*^*2*^	0.111	0.247	0.379
*df*	19	17	13
*p*	0.188	0.088	0.070

Notes: ⁎ *p* < 0.017. RBMT = Rivermead Behavioural Memory Test, HRSD = Hamilton Rating Scale for Depression, IQ = IQ by Japanese Adult Reading Test, RS = Resilience Scale, Leisure = Kinds of types of leisure experiences.

**Table 7:** Results of hierarchical regression analysis with bipolar disorder patients in JVLT.

Variables	JVLT		
	β		
	Step1	Step2	Step3
Age	−0.332	−0.425	0.137
Sex	0.066	0.066	−0.091
Duration of illness	−0.224	−0.138	−0.317
HRSD	0.154	0.248	0.338
Total sleep time		−0.195	−0.011
Physical activity		0.045	−0.038
RS			0.142
IQ			0.556*
Education			−0.260
Leisure			0.578*
**Goodness of fit of the model**			
*R*^*2*^	0.170	0.113	0.575
*df*	19	17	13
*p*	0.111	0.241	0.010

Notes: ⁎ *p* < 0.017. JVLT = Japanese Verbal Learning Test, HRSD = Hamilton Rating Scale for Depression, IQ = IQ by Japanese Adult Reading Test, RS = Resilience Scale, Leisure = Kinds of types of leisure experiences.

## 4 Discussion

In the present study, patients with BP were found to have the lower executive function, working memory, attention, processing speed, verbal fluency, story memory, and verbal memory than HC. This was even though BP patients did not differ from HC in premorbid IQ, as the Japanese Adult Reading Test estimated. Furthermore, language-related cognitive scores, such as verbal fluency and memory, were associated with more leisure experiences in patients with BP after adjusting for physical activity and sleep conditions. This is the first study to demonstrate an association between verbal fluency, verbal memory, and various leisure experiences in patients with BP, even after adjusting for daily physical activity and sleep.

Concerning the decline in BP patients’ verbal memory shown in this study, previous studies have also reported extensive but mild verbal memory deficits in BP patients in remission [44]. Patients with BP also show deficits in memory components related to frontal and temporal lobe functions [45]. Patients with BP and a longer disease duration have a smaller hippocampus than HC [46]. Furthermore, telomere length, a DNA base structure in patients with BP, is associated with hippocampal volume and episodic memory, which are associated with familial risk [47]. A recent study of the hippocampus in postmortem brains suggested that hippocampal aging in patients was associated with neurocognitive dysfunction. A complex biological clock and childhood trauma signals may influence the hippocampal aging phenomenon [48]. Previous studies have shown that childhood trauma is related to adult experiences of psychosis [49]. Increased negative affect and impulsivity have been associated with worsening BP symptoms, increased aggression, and suicidal tendencies [50]. These results suggested an important link between cognitive dysfunction and childhood experiences in patients with BP.

Previous studies have also reported moderate effect sizes for verbal fluency impairment and no significant differences between category and letter fluency. Furthermore, no significant correlation was found between the effect size of impaired verbal fluency, mean duration of illness, and mean number of mood episodes in the BP group. This indicates that impairment may have occurred much earlier [51]. These studies suggest that language-related cognitive impairments in patients with BP, such as verbal memory and verbal fluency, occur at a younger age and may be related to negative emotions and experiences, as reflected in a previous study.

Previous reports on CR and cognitive function in patients with BP suggest that a higher CR is associated with better cognitive (processing speed, working memory, verbal and visual memory, and executive attention) and psychosocial functions [11]. Additionally, educational level and pre-morbid intelligence have been shown to contribute to functional outcomes in BP patients [52]. Thus, CR may be an important factor contributing to the course and prognosis of patients with BP.

This study found an association between leisure experiences and cognitive function in patients with BP. Observational and intervention studies of healthy adults with at least two years of follow-up between 1991 and 2011 have also suggested that leisure activity has a protective effect on cognitive decline [53]. In a study by the UK Biobank, weekly participation in a sports club or gymnasium was associated with connectivity between the sensorimotor networks and lateral visual and cerebellar networks [54]. In addition, weekly visits to family and friends’ homes were associated with a greater occipital lobe volume, as measured using magnetic resonance imaging [54]. Seven general domains of daily activity (paid work, time spent with children, housework, leisure time, physical activity, formal volunteer work, and helping others) have been reported to be associated with cognitive function and well-being. Additionally, individuals with increased diversity in daily activity domains over 10 years of age have been reported to exhibit higher executive functioning [55,56]. These reports suggest that leisure experiences may be associated with cognitive function and well-being in patients with BP, related to the connectivity between the network regions regarding brain function.

A recent EEG study of older adult participants showed that leisure, a critical component of CR, is associated with cognitive function [57]. In other words, leisure may be an important protective factor of cognitive function in patients with BP. However, previous studies on the impact of CR on cognitive functioning in patients with BP have not assessed leisure as a measure separate from IQ or education [10,12,58]. In contrast to these reports, a notable feature of this study is that semi-structured in-person interviews were used to conduct individual in-depth interviews about the life experiences of each CR category. By evaluating patients with BP regarding the kinds of leisure experiences they have engaged in from childhood to the present, we can indicate a relationship between the variety of leisure experiences and cognitive function. The study showed that the greater the variety of leisure experiences, the higher the language-related cognitive function. This result is consistent with recent studies on healthy participants [55,56], which showed that diversity in daily activities is important for cognitive function. These reports suggested that language dysfunction occurs relatively early in patients with BP. However, engaging in various leisure experiences at a young age may contribute positively to brain network connectivity. Our results suggest that language dysfunction occurs fairly early in patients with BP; however, engaging in leisure experiences from a young age may be associated with brain network connectivity and potentially protective factors for language-related cognitive functions.

Moreover, in contrast to previous reports that used only questionnaires, our analysis adjusted for physical activity and sleep ratings using activity meters. Hence, objective measures of physical activity in patients with BP are highly reliable [22]. However, no significant associations existed between resilience, activity meter parameters, and cognitive function. It has been suggested that the lack of resilience in bipolar patients may be due to experiential factors such as traumatic experiences or educational deficits [14]. Further studies with larger sample sizes are needed to draw detailed conclusions regarding the relationships among resilience, sleep duration, and cognitive function.

Effective strategies for treating cognitive dysfunction in patients with BP are still under investigation [59]. Our results indicated that language-related cognitive scores such as verbal fluency and memory were positively associated with a greater variety of leisure experiences. This suggests that in addition to pharmacological treatment [59], various leisure experiences may increase the ability to maintain cognitive function as a protective factor against cognitive impairment in patients with BP.

This study has several limitations. As this was a cross-sectional study, we did not analyze the long-term effects of exercise or sleep. In this study, we chose not to include employment as a CR item owing to the early age of onset of BP and the fact that half of the participants were not employed. The decision was made to mitigate potential biases in the data. The small sample size made the correlations significant for leisure experiences related to cognitive function. However, the cross-sectional design precludes the interpretation of these causal relationships. To clarify the relationship between cognitive function and leisure experiences in patients with BP, a larger sample size should be used to analyze the frequency and kinds of years of leisure experience. The patients in the BP group exhibited remission or mild depression. However, the possible effects of medication, smoking, alcohol consumption, and physical health on cognition in patients with BP have yet to be fully examined. The association between objective neuropsychological functioning and daily behaviors (such as daily physical activity and sleep) and psychosocial factors (cognitive reserve and resilience) should be further explored in longitudinal studies with larger patient sample sizes and tightly adjusted confounding variables.

In summary, this study showed that executive function, attention, processing speed, verbal fluency, story memory, and verbal memory decreased in BP patients compared to the HC. Higher cognitive functioning, regarding verbal fluency and memory, was associated with the kinds of leisure experience types in patients with BP. In patients with bipolar disorder, susceptibility to cognitive function decline can impact learning, work, and family life. Improvements in language-related cognitive function in patients with bipolar disorder may improve their quality of life by allowing them to continue their stalled learning, work, and family lives. This cross-sectional study suggests that participation in a variety of leisure activities may prevent cognitive dysfunction in patients with bipolar disorder by increasing their cognitive reserve. However, longitudinal studies with larger sample sizes are necessary to draw conclusions. In addition, it is important to validate the present results in future longitudinal studies after ensuring statistical power to obtain true differences in effect between various measures of cognitive reserve and cognitive function.”

## Financial statement

This study was supported by Grant-in-Aid for Transformative Research Areas (A) JSPS KAKENHI Number 20H05803 and Grant-in-Aid for Scientific Research (B) JSPS KAKENHI Number 19H01761. This research was partially supported by grants from the Moonshot 10.13039/100006190Research and Development Program: grant number JPMJMS2297.

## Data availability statement

Data in the present study are available upon request from the corresponding authors. The data are not publicly available because of privacy concerns and ethical policies.

## Additional information

No additional information is available for this paper.

## CRediT authorship contribution statement

**Kuniko Sato:** Conceptualization, Investigation, Writing – original draft. **Mie Matsui:** Conceptualization, Funding acquisition, Methodology, Resources, Supervision, Writing – review & editing. **Yasuki Ono:** Data curation, Formal analysis, Methodology, Writing – original draft. **Yoshiaki Miyagishi:** Investigation, Writing – review & editing. **Makoto Tsubomoto:** Investigation, Writing – review & editing. **Nobushige Naito:** Data curation, Investigation, Writing – review & editing. **Mitsuru Kikuchi:** Formal analysis, Funding acquisition, Investigation, Writing – review & editing.

## Declaration of competing interest

The authors declare that they have no known competing financial interests or personal relationships that could have appeared to influence the work reported in this paper.
